# Association between glycemic status and the risk of acute pancreatitis: a nationwide population-based study

**DOI:** 10.1186/s13098-023-01086-x

**Published:** 2023-05-19

**Authors:** In Rae Cho, Kyung-Do Han, Sang Hyub Lee, Young Hoon Choi, Kwang Hyun Chung, Jin Ho Choi, Namyoung Park, Min Woo Lee, Woo Hyun Paik, Ji Kon Ryu, Yong-Tae Kim

**Affiliations:** 1 Department of Internal Medicine and Liver Research Institute, Seoul National University College of Medicine, Seoul, Republic of Korea; 2 Department of Statistics and Actuarial Science, Soongsil University, Seoul, Republic of Korea; 3 Department of Internal Medicine, College of Medicine, The Catholic University of Korea, Seoul, Republic of Korea; 4 Department of Internal Medicine, Soonchunhyang University Seoul Hospital, Seoul, Republic of Korea; 5 Division of Gastroenterology, Department of Medicine, Samsung Medical Center, Seoul, Republic of Korea; 6 Department of Internal Medicine, Kyung Hee University Hospital at Gangdong, Seoul, Republic of Korea

**Keywords:** Acute pancreatitis, Diabetes, Glycemic status, Risk factor

## Abstract

**Background:**

Although diabetes is reportedly associated with the occurrence of acute pancreatitis (AP), the risk of AP according to the duration and severity of diabetes is not yet clear. We aimed to investigate the risk of AP based on glycemic status and the presence of comorbidities using a nationwide population-based study.

**Methods:**

We enrolled 3,912,496 adults who underwent health examinations under the National Health Insurance Service in 2009. All participants were categorized by glycemic status as normoglycemic, impaired fasting glucose (IFG), or diabetes. Baseline characteristics and the presence of comorbidities at the time of health check-up were investigated, and the occurrence of AP was followed up until 31 December 2018. We estimated the adjusted hazard ratios (aHRs) for AP occurrence according to the glycemic status, duration of diabetes (new-onset, duration < 5 years, or ≥ 5 years), type and number of anti-diabetic medications, and presence of comorbidities.

**Results:**

During the observation period of 32,116,716.93 person-years, 8,933 cases of AP occurred. Compared with normoglycemia, the aHRs (95% confidence interval) were 1.153 (1.097–1.212) in IFG, 1.389 (1.260–1.531) in new-onset diabetes, 1.634 (1.496–1.785) in known diabetes < 5 years, and 1.656 (1.513–1.813) in patients with known diabetes aged ≥ 5 years. The presence of comorbidities associated with diabetes severity had a synergistic effect on the relationship between diabetes and AP occurrence.

**Conclusion:**

As glycemic status worsens, the risk of AP increases, and there is a synergistic effect when comorbidities coexist. To reduce the risk of AP, active control of factors that can cause AP should be considered in patients with long-standing diabetes and comorbidities.

**Supplementary Information:**

The online version contains supplementary material available at 10.1186/s13098-023-01086-x.

## Introduction

Acute pancreatitis (AP) is an acute inflammatory process of the pancreas and adjacent tissue injury with or without remote organ failure [1]. AP is a common gastrointestinal disease that requires inpatient care and is associated with significant morbidity and consumption of health care resources [2]. The global incidence of AP ranges from 23 to 43 cases per 100,000 population and has been rising in recent decades [3–5]. Gallstones and alcohol consumption are the two main causes of AP development, and other conditions such as hypertriglyceridemia, genetic factors, drugs, and anatomic variation can also cause AP [3, 6]. AP remains a clinical challenge because it can occur without an obvious etiology, and its pathogenic mechanisms are still not well understood [7].

Since only a small proportion of patients with gallstones or excessive alcohol intake develop AP [8, 9], there may be differences in individual susceptibility to AP occurrence. The mortality rate of AP is approximately 5%; however, severe complications or mortality occur in some patients. Therefore, there are also individual differences in the risk of aggravation. Thus, it may be helpful to identify high-risk groups for the occurrence and severity of AP in patients with predisposing diseases.

The prevalence of diabetes mellitus is increasing, and diabetes is a major global medical burden [10]. Diabetes patients experience various complications, depending on the duration and severity of the disease, which can cause poor quality of life and even mortality. In addition to well-known complications such as cardiovascular disease, diabetic nephropathy, and retinopathy, epidemiologic studies have shown that diabetes is associated with the occurrence of cognitive impairment, Parkinsonism, and dementia [11–13]. A relationship between diabetes and AP occurrence has also been reported in several epidemiological studies [14–16]. It is thought that obesity, dyslipidemia, and gallstones caused by diabetes are related to AP occurrence. However, the causative relationship between hyperglycemia and AP remains unclear. In addition, there is a lack of studies on the relationship between the deterioration of glycemic status and AP risk.

Therefore, we aimed to evaluate the association between glycemic status and the risk of AP occurrence. In addition, the effect of comorbidities, which are commonly associated with uncontrolled diabetes, was investigated. We used a large cohort representative of the general Korean population to determine the risk of AP according to glycemic status and the presence of comorbidities.

## Methods

### National health insurance health examination cohort

The National Health Insurance Service (NHIS) is a single healthcare insurance system that covers 97% of the total Korean population; the remaining 3% are “medical protection” beneficiaries. Information on individuals’ use of medical facilities, prescription records, and diagnostic codes configured in the form of the International Statistical Classification of Diseases and Related Health Problems, 10th revision (ICD-10) is recorded in the NHIS database [17]. In addition, NHIS provides a general health examination program for all beneficiaries aged 20 years or more and all employers of any age, which consists of anthropometry, a self-administered questionnaire on past medical history or health-related behavior, and laboratory tests. This database is considered representative of the Korean population and is used in research through anonymization and de-identification [18].

### Study participants

**Fig. 1:**
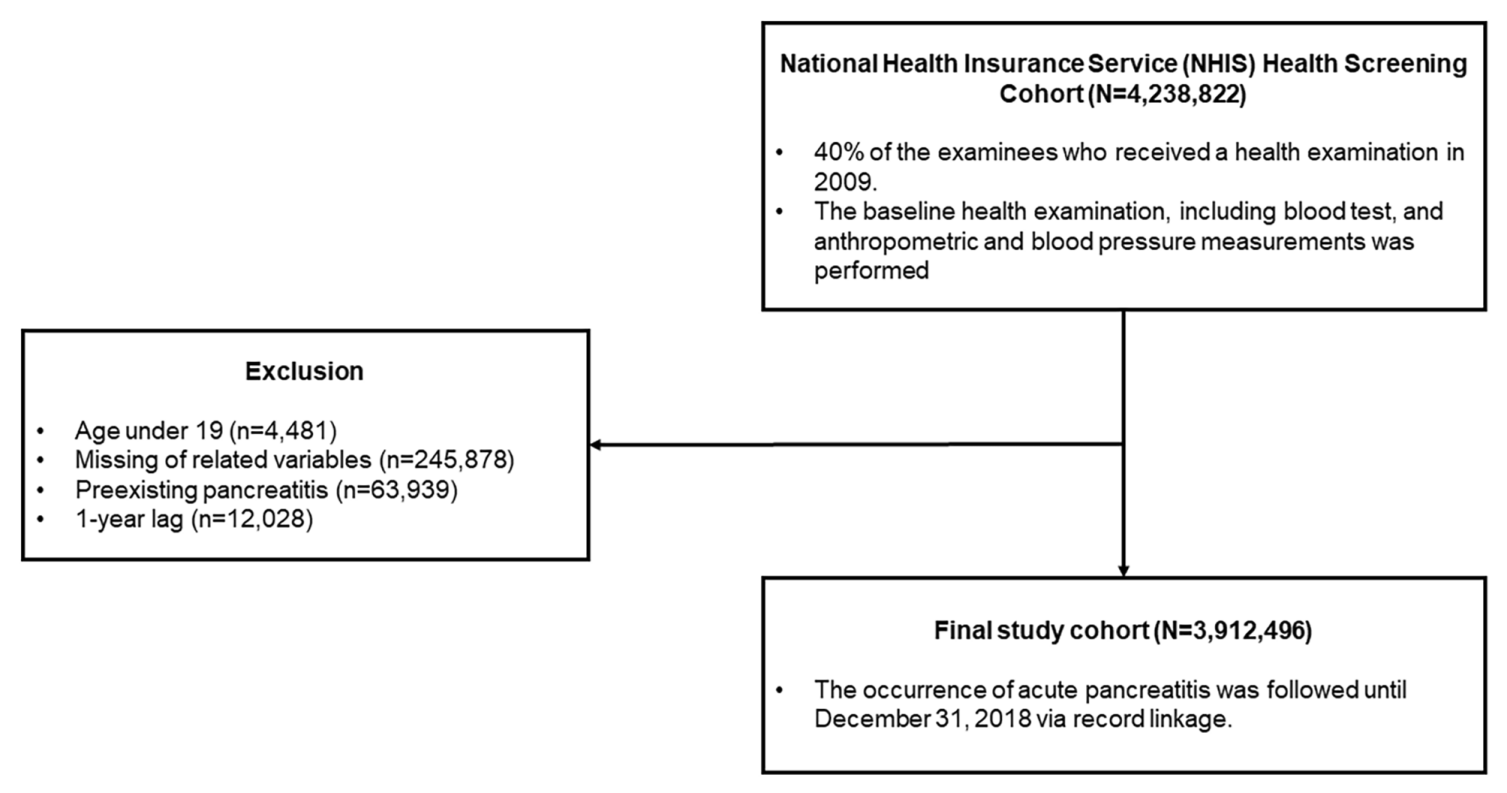
Flow diagram of the study population and enrolment

Approximately 40% of the patients (n = 4,238,822) who underwent the NHIS health examination in 2009 were sampled through the standardization process and constituted the study cohort. We excluded the following patients from the analysis: (1) aged 19 years or younger (n = 4,481), (2) those with a previous history of AP from 2002 to the time of receiving a health check-up (washout, n = 63,939), (3) participants who were diagnosed with AP within a year of health check-up (1-year lag period, n = 12,028), and (4) those with missing data (n = 245,878). As a result, 3,912,496 participants were included in the analysis. The flowchart of the study population is shown in Fig. 1.

Participants’ diabetes and glucose tolerance status were categorized into groups according to ICD-10 codes (E11.x–E14.x), prescription records (oral and/or injectable antidiabetic medications), and fasting glucose measurements during health screening. This definition was based on the consensus of relevant findings widely used in previous studies [12, 19]. The participants were classified based on glycemic status as normoglycemia (fasting glucose < 100 mg/dL), impaired fasting glucose (IFG, fasting glucose 100–125 mg/dL), and diabetes. The diagnosis of IFG and diabetes was based on the criteria of the American Diabetes Association [20]. The duration of diabetes (new-onset diabetes, known diabetes with a duration < 5 years, and diabetes duration ≥ 5 years) was identified based on the time point from the first prescription date of anti-diabetic medication to the health examination date. All participants were followed up until 31 December 2018, and the median observation period was 8.31 (interquartile range, 8.11–8.57) years.

### Clinical variables

During the health check-up, physical examinations of the participants were performed by medical professionals, and height, weight, waist circumference, and systolic and diastolic blood pressure were assessed. Detailed information about the participants’ lifestyles was obtained through standardised self-reported questionnaires. Participants were classified as non-smokers, former smokers, or current smokers based on smoking status. Based on the quantity of daily alcohol intake, participants were categorized into three groups non-drinkers, mild drinkers (0–30 g ethanol/day), and heavy drinkers (≥ 40 g ethanol/day). Regular physical activity was defined as the performance of strenuous exercise for at least 20 min (once per week)[21]. Comorbidities (gallstone disease, hypertension, diabetes, dyslipidemia, coronary artery disease, and cerebrovascular disease) were identified by combining data from past medical history questionnaires, ICD-10 codes, and prescription databases. Blood samples were collected after overnight fasting, and serum glucose, total cholesterol, triglyceride, high-density lipoprotein (HDL) cholesterol, low-density lipoprotein (LDL) cholesterol, aspartate aminotransferase (AST), alanine aminotransferase (ALT), gamma-glutamyl transferase (GGT), and creatinine levels were measured. The eGFR was calculated using the Modification of Diet in Renal Disease (MDRD) equation. Chronic kidney disease was defined as eGFR < 60 mL/min/1.73 m^2^. All these clinical variables were collected during health check-ups and were used as baseline characteristics.

### Identification of acute pancreatitis and comorbidity status

AP was diagnosed when a patient was discharged from the hospital with ICD-10 code of AP (K85.x) before the health check-up and during the follow-up period. Baseline comorbidities were identified as gallstone disease (ICD-10 codes K80.x, K81.x, K83.0x, and K85.1x), hypertension (ICD-10 codes I10.x–I13.x, I15.x, and treatment with antihypertensive medications, systolic/diastolic blood pressure ≥ 140/90 mmHg, or both), dyslipidemia (ICD-10 code E78.x with lipid-lowering agents, or serum total cholesterol ≥ 240 mg/dL, or both), and chronic kidney disease (CKD) (eGFR < 60 mL/min/1.73 m^2^ [27]). Coronary artery disease (ICD-10 codes I20.x-I25x) and cerebrovascular disease (ICD-10 codes I61.x-I63.x, I65.x, I66.x, I67.0x, I67.1x, I67.5x-I67.9x, I68.1x, I68.2x, I68.8x, I69.x, G45.0x-G45.2x, G45.4x, G45.8x, G45.9x, and G46x) were defined according to the ICD-10 codes.

### Statistical analysis

Continuous variables are expressed as mean ± standard deviation or median and inter-quartile range (IQR) for variables that did not follow a normal distribution. Categorical variables are presented as numbers and percentages. To compare the difference among the groups, the chi-squared test was used for categorical variables, one-way analysis of variance (ANOVA) for normally distributed continuous variables, and the Kruskal–Wallis test for not normally distributed continuous variables. The incidence rates of AP were calculated by dividing the number of incident cases in each group by the total follow-up period per 1000 person-years (PY). The Cox proportional hazards model was used to evaluate adjusted hazard ratios (aHRs) and 95% CIs for AP. A multivariable-adjusted proportional hazards model was applied: (i) Model 1 was adjusted for age and sex, and (ii) Model 2 was further adjusted for smoking, alcohol consumption, gallstone, regular physical activity, income status, hypertension, dyslipidemia, hypertriglyceridemia, and chronic kidney disease. Statistical analyses were performed using Statistical Analysis System (SAS) version 9.4 (SAS Institute Inc., Cary, NC, USA), and a P value less than 0.05 was considered statistically significant.

## Results

### Baseline characteristics

The baseline characteristics of the study population are summarized in Table 1. Among a total of 3,912,496 participants, the percentages of those in the groups with normoglycemia, IFG, and diabetes were 68.5% (n = 2,697,854), 22.8% (n = 890,822), and 8.7% (n = 341,820), respectively (Table 1). The diabetes status worsened with increasing age. The prevalence of comorbidities also increased with the deterioration of glycemic status and diabetes duration. Patients with diabetes were more obese and had higher systolic blood pressure than those without diabetes or IFG. The rates of current smokers and participants with high alcohol consumption were highest in the new-onset diabetes group. The proportion of patients treated with insulin was higher in the diabetes duration ≥ 5 years group than in the < 5 years group (8.03% vs. 3.63%).

**Table 1:** Baseline characteristics of the study population

Variables	No diabetes n = 2,679,854	IFG n = 890,822	Diabetes n = 341,820			P-value
			New onset DM n = 116,806	DM duration <5 years n = 115,635	DM duration ≥ 5 years n = 109,379	
Age	45.02 ± 13.81	49.69 ± 13.27	51.86 ± 12.68	58.34 ± 11.1	61.98 ± 9.96	< 0.001
Male Sex	1,371,370 (51.17)	552,159 (61.98)	82,983 (71.04)	67,116 (58.04)	59,618 (54.51)	< 0.001
*Smoking status*						< 0.001
Never smoker	1,656,134 (61.8)	486,916(54.66)	54,567(46.72)	66,847(57.81)	69,001(63.08)	
Ex-smoker	341,490 (12.74)	156,332(17.55)	21,381(18.3)	21,627(18.7)	19,569(17.89)	
Current smoker	682,230 (25.46)	247,574(27.79)	40,858(34.98)	27,161(23.49)	20,809(19.02)	
*Alcohol consumption*						< 0.001
None	1,403,799(52.38)	423,311(47.52)	50,850(43.53)	70,494(60.96)	73,660(67.34)	
Mild	1,093,600(40.81)	374,694(42.06)	49,447(42.33)	34,833(30.12)	28,400(25.96)	
Heavy	182,455(6.81)	92,817(10.42)	16,509(14.13)	10,308(8.91)	7,319(6.69)	
*Regular physical activity*						
Yes	457,948 (17.09)	169,013 (18.97)	22,175 (18.98)	25,632 (22.17)	26,589 (24.31)	< 0.001
*Comorbidity*						
Gallstone disease	37,518(1.4)	16,302(1.83)	2,032(1.74)	6,106(5.28)	5,688(5.2)	< 0.001
Hypertension	547,721(20.44)	307,027(34.47)	52,688(45.11)	75,148(64.99)	75,981(69.47)	< 0.001
Coronary artery disease	84,773(3.16)	43,627(4.9)	6,238(5.34)	16,425(14.2)	20,011(18.3)	< 0.001
Cerebrovascular disease	74,787(2.79)	35,382(3.97)	5,089(4.36)	12,531(10.84)	15,269(13.96)	< 0.001
Chronic kidney disease	156,691(5.85)	70,149(7.87)	10,161(8.7)	13,251(11.46)	19,699(18.01)	< 0.001
Dyslipidemia	373,685(13.94)	198,273(22.26)	33,251(28.47)	55,709(48.18)	51,538(47.12)	< 0.001
*Body measurements*						
BMI (m^2^/kg)	23.32 ± 3.15	24.36 ± 4.11	24.99 ± 3.41	25.37 ± 3.33	24.7 ± 3.15	< 0.001
Waist circumference (cm)	78.83 ± 9.37	82.39 ± 8.99	84.97 ± 8.88	86.17 ± 8.82	85.35 ± 9.06	< 0.001
Systolic BP (mmHg)	120.48 ± 14.5	125.8 ± 15.09	129.78 ± 16.06	128.73 ± 15.6	129.04 ± 15.91	< 0.001
Diastolic BP (mmHg)	75.28 ± 9.85	78.34 ± 10.14	80.57 ± 10.52	79.08 ± 10.08	77.63 ± 9.93	< 0.001
*Laboratory findings*						
Fasting glucose (mg/dL)	87.51 ± 7.74	107.78 ± 6.56	154.47 ± 41.76	138.26 ± 51.18	147.19 ± 54.33	< 0.001
Total cholesterol (mg/dL)	192.71 ± 39.48	202.17 ± 44.3	207.82 ± 47.81	195.67 ± 47.45	188.68 ± 46.02	< 0.001
HDL cholesterol (mg/dL)	57.19 ± 33.07	55.64 ± 31.66	53.81 ± 34.55	52.38 ± 32.29	52.09 ± 31.96	< 0.001
LDL cholesterol (mg/dL)	121.27 ± 230.32	124.16 ± 198.69	119.39 ± 118.12	111.92 ± 98.88	107.53 ± 78.71	< 0.001
Triglyceride (mg/dL)^§^	101 (70–150)	125 (86–185)	159 (106–242)	142 (100–206)	135 (95–195)	< 0.001
AST^§^	22 (18–27)	24 (20–30)	26 (21–34)	25 (20–32)	23 (19–29)	< 0.001
ALT^§^	19 (14–27)	22 (16–32)	27 (19–41)	25 (18–37)	23 (17–32)	< 0.001
r-GTP^§^	21 (15–34)	28 (18–49)	41 (24–75)	32 (21–56)	27 (18–43)	< 0.001

* Abbreviations: IFG, impaired fasting glucose; DM, diabetes mellitus; BMI, body mass index; BP, blood pressure; HDL, high density lipoprotein; LDL, low density lipoprotein ;AST, aspartate aminotransferase ;ALT, alanine aminotransferase; r-GTP, gamma glutamyl transpeptidase

** Data were expressed as the mean ± standard deviation, or n (%)

§ Non-normally distributed variables were expressed as the median (interquartile range)

### Incidence of acute pancreatitis according to the glycemic status

**Fig. 2:**
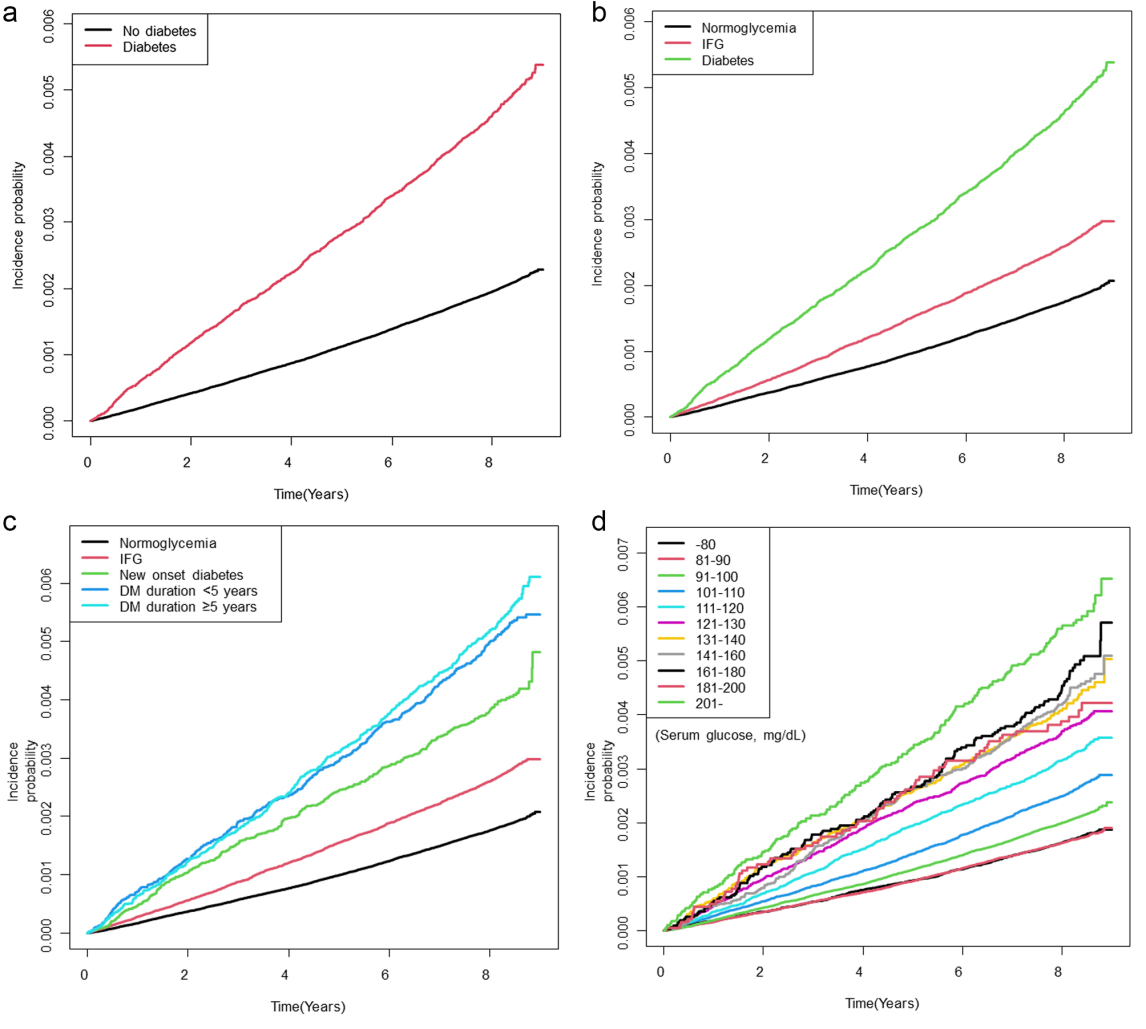
(A) Comparison of cumulative incidence of acute pancreatitis between diabetes and non-diabetes subjects, (B) normoglycemic subjects, impaired fasting glucose (IFG), and diabetes patients, and (C) normoglycemic subjects, impaired fasting glucose, new-onset diabetes, diabetes duration < 5years and ≥ 5years. and (D) Cumulative incidence of acute pancreatitis according to the serum glucose level

The total observation period was 32,116,716.93 PY, and 8,933 cases of AP occurred (Table 2). The incidences of AP in non-diabetes and diabetes participants were 0.25 and 0.59 per 1,000 PY, respectively. The incidence rate significantly increased with the deterioration of the glycemic status. The cumulative incidence of AP in each group was calculated using the Kaplan-Meier curve (Fig. 2A-C).

**Table 2:** Incidence rates and multivariate adjusted HRs of acute pancreatitis according to glycemic status at various time point from the index health examination

Glycemic status	n	Events (n)	PYs	Incidence	Adjusted hazard ratio - Model 2 (95% CI)			
				per 1,000 PY				
					1 year	3 years	5 years	up to 9 years ^§^
*Non-DM vs. DM*								
Non-DM (normal + IFG)	3,570,676	7,334	29,398,294.94	0.24947	1(Ref.)	1(Ref.)	1(Ref.)	1(Ref.)
DM	341,820	1,599	2,718,421.99	0.58821	1.733 (1.479–2.03)	1.664 (1.514–1.829)	1.558 (1.447–1.678)	1.479 (1.398–1.565)
*Glycemic status*								
Normal	2,679,854	4,921	22,105,281.33	0.22262	1(Ref.)	1(Ref.)	1(Ref.)	1(Ref.)
IFG	890,822	2,413	7,293,013.61	0.33086	1.209 (1.042–1.403)	1.113 (1.02–1.214)	1.122 (1.051–1.199)	1.152 (1.096–1.211)
DM	341,820	1,599	2,718,421.99	0.58821	1.871 (1.578–2.218)	1.734 (1.568–1.918)	1.630 (1.507–1.763)	1.567 (1.477–1.662)

* Abbreviations: IFG, impaired fasting glucose; DM, diabetes mellitus; PY, person-year

§ end of follow-up period

HR values were greater in patients with diabetes than those without diabetes, even after adjusting for confounding factors. Patients had an approximately 1.5-times higher risk of developing AP than normoglycemic participants. In addition, the risk of AP occurrence increased with the deterioration of the glycemic status. The aHR (95% CI) for AP was 1.152 (1.096–1.211) in those with IFG and 1.567 (1.477–1.662) in those with diabetes. This relationship between glycemic status and AP risk was consistently observed during the follow-up period from the index date of health examination.

### Incidence of acute pancreatitis according to the duration of diabetes

Comparing the subgroups according to glucose tolerance and duration of diabetes, the aHRs for AP were higher in patients with known diabetes than in those with IFG or new-onset diabetes (Supplemental Table 1). There was no remarkable difference among patients with known diabetes when the duration of diabetes was divided by a duration of 5 years. The aHR (95% CI) for AP was 1.153 (1.097–1.212) in IFG, 1.389 (1.260–1.531) in new-onset diabetes, 1.634 (1.496–1.785) in known diabetes < 5 years, and 1.656 (1.513–1.813) in patients with known diabetes aged ≥ 5 years. The statistical data (p-values) for comparisons between the subgroups are presented in Supplemental Table 2.

In the subgroup analysis based on the type and number of anti-diabetic medications (one oral medication, 2 or more oral medications, and insulin use), it was confirmed that insulin users had a high risk of AP occurrence in diabetes patients (Supplemental Table 3). In particular, insulin users in the diabetes < 5 years group had a significantly higher AP risk. This result suggests that the rapid deterioration of glycemic status increases the AP risk.

### Incidence of acute pancreatitis according to the serum glucose level

HbA1c, which reflects average blood sugar levels over the past 3 months, could not be confirmed due to the limitations of the health examination data. Therefore, according to the increase in serum glucose levels at health examination, the risk of AP was analyzed (Supplemental Table 1). The aHR (95% CI) was the highest in the group with serum glucose > 200 mg/dL (1.843, 1.580–2.150), and there was a statistically significant difference in the cumulative incidence (Fig. 2D). Although the increasing trend of aHR was attenuated compared to the unadjusted HR, aHRs gradually increased as the serum glucose level increased, except for one group (serum glucose level 181–200 mg/dL), where the number of participants was relatively small (n = 18,065).

### Incidence of acute pancreatitis according to glycemic status and comorbidities

**Fig. 3:**
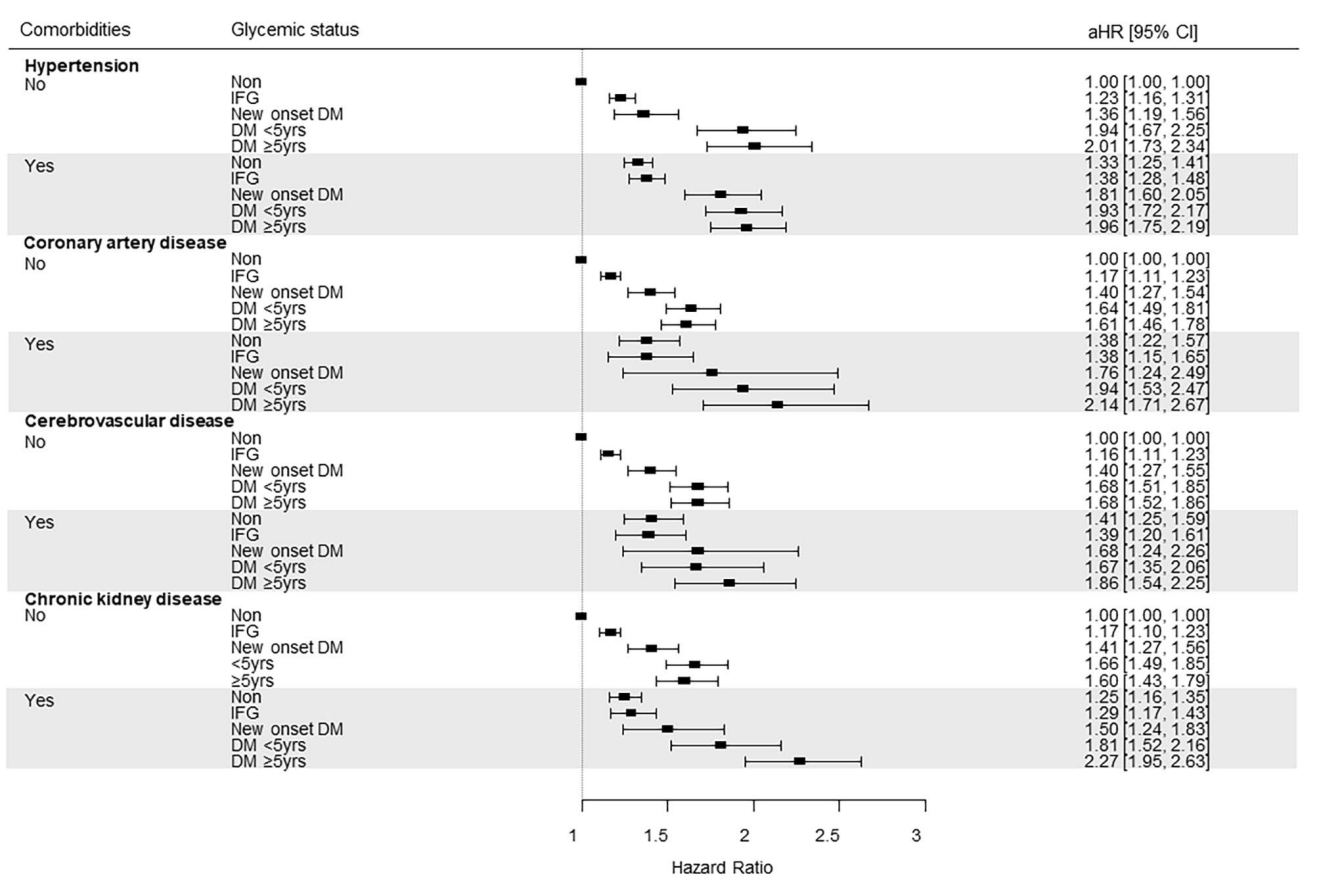
Multivariate aHRs of acute pancreatitis according to glycemic status and presence of comorbidities. (aHRs were calculated by adjusting for age, sex, smoking, alcohol consumption, gallstone, regular physical activity, income status, hypertension, dyslipidemia, hypertriglyceridemia, and chronic kidney disease. Each dot plot represents the aHR value for each group)

The AP risk according to glycemic status and the presence of comorbidities is summarized in Fig. 3. The presence of comorbidities such as hypertension, coronary artery disease, cerebrovascular disease, and chronic kidney disease was associated with an increased risk of AP.

The effect of comorbidity on the increase in AP risk differs according to the duration of diabetes and type of comorbidity. In the absence of comorbidities, the aHRs for AP in patients with known diabetes < 5 years were similar to those in patients with diabetes ≥ 5 years. However, in patients with coronary artery disease, cerebrovascular disease, and chronic kidney disease, the aHR increased as the duration of diabetes increased. For example, in the absence of CKD, the aHRs (95% CI) for AP in patients with diabetes duration < 5 years and ≥ 5 years were 1.660 (1.490–1.850) and 1.600 (1.430–1.790), respectively. However, in the presence of CKD, the aHRs (95% CIs) for AP were 1.810 (1.520–2.160) and 2.270 (1.950–2.630), respectively.

When participants with the same glycemic status were compared, AP risk was higher in those with comorbidities. In addition, the AP was higher in patients with diabetes and comorbidities than in patients who had a longer duration of diabetes without comorbidities. For example, when comparing patients with diabetes ≥ 5 years without comorbidities and diabetes < 5 years with comorbidities, the aHR for AP was higher in patients with comorbidities. In patients without coronary artery disease, the aHR (95% CI) for AP in patients with diabetes duration ≥ 5 years was 1,610 (1.460–1.780), while the aHR in patients with coronary artery disease and < 5 years duration of diabetes was 1.940 (1.530–2.470). Similar results have been observed in patients with chronic kidney diseases.

When the risk of AP was compared in patients with diabetes without comorbidities and patients without diabetes but with hypertension, coronary artery disease, cerebrovascular disease, or chronic kidney disease, the aHR was higher in patients with diabetes without comorbidities. The aHR (95% CI) for AP in hypertension patients without diabetes was 1.330 (1.250–1.410), whereas the aHR (95% CI) of diabetes patients without hypertension was 1.940 (1.670–2.250; <5 years), and 2.010 (1.730–2.340; ≥5 years), respectively.

## Discussion

In this nationwide population-based study, we found that deterioration of glycemic status is associated with an increased risk of AP, and the presence of comorbidities such as hypertension, coronary artery disease, cerebrovascular disease, and chronic kidney disease resulted in synergistic effects on the risk of AP occurrence. Patients with diabetes and no comorbidities had a higher risk of AP than those with comorbidities but were nondiabetic. However, when comorbidities were present in patients with diabetes, the risk of developing AP was higher than that in patients without comorbidities. To the best of our knowledge, this study is the largest to find that the risk of AP occurrence is related to glycemic status, duration of diabetes, and comorbidities.

Several previous studies have found that diabetes increases the risk of AP,[14–16] and several epidemiological features could explain the association of diabetes with AP. Patients with diabetes are more likely to have gallstones and dyslipidemia that could result in AP occurrence [22, 23]. In addition, patients with diabetes have behaviors that increase the risk of pancreatitis, such as alcohol and tobacco use [24, 25]. There are also reports that there is an association between the use of antidiabetic medication and AP risk [26, 27].

In addition to epidemiological features, there is a potential mechanism by which hyperglycemia itself may contribute to the development of AP. Hyperglycemia causes oxidative stress, which in turn stimulates the generation of inflammatory mediators and the production of reactive oxygen species [28]. These conditions can predispose and aggravate AP [29, 30]. In addition, chronic vascular inflammation caused by diabetes may decrease the microcirculation of the pancreas and increase the risk of ischemic AP under certain conditions [31, 32]. Considering that many years of hyperglycemic status precede the diagnosis of type 2 diabetes [33], it can be understood that participants with IFG and new-onset diabetes, who have been exposed to hyperglycemic stress for several years, have a higher risk of AP compared to those with normoglycemia.

Several metabolic conditions are known to be associated with the severity of AP. It has been reported that the presence of metabolic syndrome, non-alcoholic fatty liver disease, and large visceral adipose tissue volume can be predictors of severe pancreatitis [34–36]. It has also been reported that diabetes increases the risk of severe AP and mortality [37, 38]. In this aspect, further research is possible on the association between severe AP and diabetes and its comorbidity. This study could not confirm the severity of AP, and this can be a future research project.

Several factors can explain the synergistic effect of comorbidities on the increase in AP risk. In patients with diabetes, intensive glycemic control is associated with a reduced risk of microvascular complications [39]. Diabetic nephropathy is one of the microvascular complications; therefore, the presence of CKD could be related to the longer duration of diabetes and poor glycemic control. Similarly, the presence of coronary artery and cerebrovascular diseases may also reflect macrovascular complications in patients with diabetes.

The comorbidities themselves could also contribute to an increase in AP risk. Cardiovascular diseases share risk factors with AP, including alcohol consumption and obesity. It can also reduce splanchnic and pancreatic blood flow that causes local ischemia and activates the renin-angiotensin system, which is related to angiotensin-II level, a proinflammatory hormone in the pancreas [40]. CKD can cause an increase in gastrointestinal hormones such as cholecystokinin, a gastric inhibitory polypeptide that causes hypersecretion of pancreatic enzymes, eventually leading to impaired pancreatic function [41, 42]. Patients on peritoneal dialysis are known to have a significantly increased risk for AP due to contributing factors such as alterations in serum calcium and parathyroid hormone levels, bacterial and viral infections, and toxic substances in the PD dialysate [43–45].

There are reports that diabetes could develop after the onset of exocrine pancreatic diseases [46, 47]. In recent studies, it has been reported that the proportion of patients with newly diagnosed diabetes after the onset of AP ranges from 11 to 23% [48, 49]. Islet cell loss due to extensive pancreatic necrosis, persistent inflammatory response, and disruption of the insulin-incretin axis have been suggested as the pathophysiology of diabetes following AP [50]. Based on this hypothesis, it can be thought that AP may accelerate the aggravation of diabetes when AP occurs in IFG participants. In addition, there is a possibility of a further decrease in endocrine pancreatic function when AP occurs in patients with diabetes. Thus, strict sugar control and prevention of comorbidities are strongly recommended in patients with diabetes to prevent further deterioration of the glycemic status due to AP.

We attempted to overcome the possibility of confounding factors due to the nature of this study through additional analysis. The “healthy-user effect” is an inevitable bias in observational studies based on health examination data. Previous study has suggested identifying active comparators and improving statistical adjustment to minimize this kind of bias. [51]. However, this study cohort was constituted based on the NHIS health examination data, and therefore it was not possible to identify a comparator group who did not receive health examination and to confirm their clinical variables, lifestyle, and preventive therapy. Instead, to minimize this kind of bias, we adjusted healthy behavior-related parameters (regular physical activity) and income status as compounding factors. In the long-term follow-up study, the effects of the time-varying variables measured at baseline could change over time. As this can be an important confounding factor in interpreting the results, we analyzed the risk of AP occurrence according to the glycemic status at 1-, 3-, and 5-year from the health examination. Consequently, although the magnitude of the aHR values decreased over time, the significant relationship between the deterioration of glycemic status and AP risk was maintained.

This study has also limitations. First, we could not adjust for all risk factors of AP, despite adjusting for the well-known factors. The prevalence of gallstones in patients with diabetes is reportedly high. However, it was difficult to accurately investigate the prevalence of gallstones (especially asymptomatic gallstones) from medical history questionnaires and ICD-10 codes. Therefore, it is possible that the prevalence of gallstones was underestimated. Additionally, other risk factors, such as hypercalcemia, could not be adjusted due to the limitations of the database. To address this problem, we intend to investigate further with a prospective cohort study. Second, because this study was based on claims and health check-up data, the sampling is likely to be inaccurate, in contrast to that in a directly sampled cohort. Also, owing to the nature of national health screening, it was difficult to confirm information such as HbA1c and response to antidiabetic medications that were not included in the laboratory examination or questionnaire. Further, because we confirmed only the occurrence of AP with the discharge data, it was not possible to investigate the severity and mortality of AP and the effect of glycemic status on the recurrence of AP. Last, despite multivariable adjustment and additional subgroup analyses, the influence of bias due to the nature of the study design cannot be completely ruled out. Further studies reflecting health-seeking behaviors and time-varying effects of clinical variables are needed.

Despite its limitations, this study has advantages, as it was based on a large cohort representative of the entire population of Korea. To minimize the limitations of claim-based research, this study investigated the detailed characteristics of the subjects based on clinical information and adjusted for as many variables as possible when constructing a proportional hazards model. In addition to the large number of subjects, a median of 8.4 years of observation allowed us to examine the causality between glycemic status and AP more rigorously.

In conclusion, our nationwide population-based study clearly suggests that deterioration of glycemic status is associated with AP and that there is a synergistic effect on AP occurrence when comorbidities coexist. To reduce the risk of AP, lifestyle modifications and treatment of the predisposing disease should be recommended in patients with long-standing diabetes with comorbidities.

## Electronic supplementary material

Below is the link to the electronic supplementary material.

**Additional File 1Supplemental Table 1** Incidence rates and multivariate aHRs of acute pancreatitis according to glycemic status, duration of diabetes, and the serum glucose level. **Supplemental Table 2** Statistical data (p-values) for comparison of the aHR for acute pancreatitis between the subgroups classified according to the glycemic status. **Supplemental Table 3** Subgroup analyses based on the type and number of anti-diabetic medications in diabetes patients.

## Data Availability

All data relevant to the study are included in the article or uploaded as online supplemental information.
