# Supplementary material for: Association between glycemic status and the risk of acute pancreatitis: a nationwide population-based study
Source: Diabetol Metab Syndr. 2023 May 19;15:104. doi: 10.1186/s13098-023-01086-x (PMC10197233; doi:10.1186/s13098-023-01086-x)
Supplement: Supplementary file 1 — Additional File 1Supplemental Table 1 Incidence rates and multivariate aHRs of acute pancreatitis according to glycemic status, duration of diabetes, and the serum glucose level. Supplemental Table 2 Statistical data (p-values) for comparison of the aHR for acute pancreatitis between the subgroups classified according to the glycemic status. Supplemental Table 3 Subgroup analyses based on the type and number of anti-diabetic medications in diabetes patients. [file 13098_2023_1086_MOESM1_ESM.docx]

**Supplemental Table 1.**  Incidence rates and multivariate aHRs of acute pancreatitis according to glycemic status, duration of diabetes, and the serum glucose level

| Glycemic status | n | Events (n) | PYs | Incidence  per 1,000 PY | Hazard ratio (95% CI) | | |
| --- | --- | --- | --- | --- | --- | --- | --- |
|  |  |  |  |  | Unadjusted | Adjusted - Model 1 | Adjusted - Model 2 |
| *Diabetes subgroups* |  |  |  |  |  |  |  |
| Normal | 2,679,854 | 4,921 | 22,105,281.33 | 0.22262 | 1(Ref.) | 1(Ref.) | 1(Ref.) |
| IFG | 890,822 | 2,413 | 7,293,013.61 | 0.33086 | 1.487(1.416-1.561) | 1.240(1.180-1.303) | 1.153(1.097-1.212) |
| New onset DM | 116,806 | 456 | 939,643.64 | 0.48529 | 2.185(1.985-2.405) | 1.652(1.500-1.820) | 1.389(1.26-1.531) |
| DM duration <5 years | 115,635 | 578 | 925,130.71 | 0.62478 | 2.805(2.574-3.058) | 1.905(1.745-2.080) | 1.634(1.496-1.785) |
| DM duration≥5 years | 109,379 | 565 | 853,647.64 | 0.66187 | 2.981(2.732-3.252) | 1.870(1.709-2.045) | 1.656(1.513-1.813) |
| *Serum Glucose level, mg/dL* |  |  |  |  |  |  |  |
| ~ 80 | 447,190 | 771 | 3,690,029.5 | 0.20894 | 1(Ref.) | 1(Ref.) | 1(Ref.) |
| 81~90 | 1,043,592 | 1,780 | 8,613,399.87 | 0.20665 | 0.989(0.909-1.076) | 0.939(0.863-1.022) | 0.949(0.872-1.033) |
| 91~100 | 1,223,970 | 2,552 | 10,072,644.62 | 0.25336 | 1.213(1.119-1.315) | 1.045(0.964-1.133) | 1.032(0.952-1.119) |
| 101~110 | 614,540 | 1,608 | 5,036,767.88 | 0.31925 | 1.529(1.403-1.666) | 1.193(1.094-1.300) | 1.129(1.035-1.232) |
| 111~120 | 257,207 | 838 | 2,092,072.63 | 0.40056 | 1.919(1.740-2.116) | 1.361(1.233-1.502) | 1.233(1.116-1.362) |
| 121~130 | 117,992 | 442 | 952,538.33 | 0.46402 | 2.223(1.978-2.499) | 1.48(1.316-1.666) | 1.295(1.151-1.457) |
| 131~140 | 58,746 | 247 | 472,491.27 | 0.52276 | 2.505(2.171-2.891) | 1.619(1.402-1.871) | 1.4(1.211-1.618) |
| 141~160 | 62,324 | 270 | 498,990.15 | 0.54109 | 2.593(2.258-2.979) | 1.655(1.439-1.903) | 1.418(1.233-1.631) |
| 161~180 | 30,772 | 145 | 245,408.14 | 0.59085 | 2.833(2.372-3.383) | 1.804(1.509-2.156) | 1.509(1.262-1.804) |
| 181~200 | 18,065 | 70 | 143559.07 | 0.4876 | 2.338(1.831-2.987) | 1.518(1.188-1.940) | 1.258(0.985-1.607) |
| 200~ | 38,098 | 210 | 298815.49 | 0.70277 | 3.375(2.898-3.932) | 2.285(1.960-2.664) | 1.843(1.580-2.150) |

* Abbreviations: IFG, impaired fasting glucose; DM, diabetes mellitus; PY, person-year

**Supplemental Table 2.** Statistical data (p-values) for comparison of the aHR for acute pancreatitis between the subgroups classified according to the glycemic status.

| **Unadjusted** | | | | | |
| --- | --- | --- | --- | --- | --- |
|  | Normal | IFG | New onset DM | DM duration <5 years | DM duration≥5 years |
| Normal | - | <.0001 | <.0001 | <.0001 | <.0001 |
| IFG | <.0001 | - | <.0001 | <.0001 | <.0001 |
| New onset DM | <.0001 | <.0001 | - | <.0001 | <.0001 |
| DM duration <5 years | <.0001 | <.0001 | <.0001 | - | 0.3057 |
| DM duration≥5 years | <.0001 | <.0001 | <.0001 | 0.3057 | - |
| **Adjusted - Model 1** | | | | | |
|  | Normal | IFG | New onset DM | DM duration <5 years | DM duration≥5 years |
| Normal | - | <.0001 | <.0001 | <.0001 | <.0001 |
| IFG | <.0001 | - | <.0001 | <.0001 | <.0001 |
| New onset DM | <.0001 | <.0001 | - | 0.0232 | 0.0509 |
| DM duration <5 years | <.0001 | <.0001 | 0.0232 | - | 0.7481 |
| DM duration≥5 years | <.0001 | <.0001 | 0.0509 | 0.7481 | - |
| **Adjusted - Model 2** | | | | | |
|  | Normal | IFG | New onset DM | DM duration <5 years | DM duration≥5 years |
| Normal | - | <.0001 | <.0001 | <.0001 | <.0001 |
| IFG | <.0001 | - | <.0001 | <.0001 | <.0001 |
| New onset DM | <.0001 | <.0001 | - | 0.0099 | 0.0057 |
| DM duration <5 years | <.0001 | <.0001 | 0.0099 | - | 0.8214 |
| DM duration≥5 years | <.0001 | <.0001 | 0.0057 | 0.8214 | - |

* Abbreviations: IFG, impaired fasting glucose; DM, diabetes mellitus

**Supplemental Table 3.** Subgroup analyses based on the type and number of anti-diabetic medications in diabetes patients.

|  | n | Events (n) | PYs | Incidence | Hazard ratio (95% CI) | | |
| --- | --- | --- | --- | --- | --- | --- | --- |
|  |  |  |  | per 1,000 PY | Unadjusted | Adjusted - | Adjusted - |
|  |  |  |  |  |  | Model 1 | Model 2 |
| ***All DM patients*** | 225,014 |  |  |  |  |  |  |
| 1 oral medication | 61,390 | 287 | 490205 | 0.5855 | 1(Ref.) | 1(Ref.) | 1(Ref.) |
| ≥2 oral medications | 132,138 | 649 | 1051432 | 0.6173 | 1.055(0.915,1.218) | 1.061(0.919,1.224) | 1.048(0.908,1.211) |
| Insulin use | 31,486 | 207 | 237142 | 0.8729 | 1.494(1.243,1.797) | 1.511(1.257,1.816) | 1.454(1.207,1.751) |
| ***DM duration <5 years*** | 115,635 |  |  |  |  |  |  |
| 1 oral medication | 45,801 | 203 | 368649 | 0.5534 | 1(ref.) | 1(ref.) | 1(ref.) |
| ≥2 oral medications | 59,334 | 286 | 476380 | 0.6004 | 1.086(0.899,1.31) | 1.086(0.899,1.312) | 1.094(0.905,1.322) |
| Insulin use | 10,500 | 89 | 80102 | 1.1111 | 2.000(1.541,2.596) | 2.011(1.549,2.611) | 1.891(1.452,2.462) |
| ***DM duration≥5 years*** | 109,379 |  |  |  |  |  |  |
| 1 oral medication | 15,589 | 84 | 121556 | 0.6910 | 1(ref.) | 1(ref.) | 1(ref.) |
| ≥2 oral medications | 72,804 | 363 | 575052 | 0.6312 | 0.994(0.783,1.261) | 1.025(0.808,1.302) | 0.999(0.786,1.268) |
| Insulin use | 20,986 | 118 | 157040 | 0.7514 | 1.210(0.913,1.604) | 1.260(0.950,1.670) | 1.232(0.926,1.639) |
